# Nanostructured hybrid ZnO thin films for energy conversion

**DOI:** 10.1186/1556-276X-6-384

**Published:** 2011-05-16

**Authors:** Mónica Moya, Anura Priyajith Samantilleke, Miquel Mollar, Bernabé Marí

**Affiliations:** 1 Department de Física Aplicada-IDF, Universitat Politècnica de València, Camí de Vera s/n, 46022, València, Spain; 2 Centro de Física, Universidade do Minho, Braga, 4700-057 Braga, Portugal

## Abstract

We report on hybrid films based on ZnO/organic dye prepared by electrodeposition using tetrasulfonated copper phthalocyanines (TS-CuPc) and Eosin-Y (EoY). Both the morphology and porosity of hybrid ZnO films are highly dependent on the type of dyes used in the synthesis. High photosensitivity was observed for ZnO/EoY films, while a very weak photoresponse was obtained for ZnO/TS-CuPc films. Despite a higher absorption coefficient of TS-CuPc than EoY, in ZnO/EoY hybrid films, the excited photoelectrons between the EoY levels can be extracted through ZnO, and the porosity of ZnO/EoY can also be controlled.

## Introduction

Replacing the conventional TiO_2 _nanocrystalline (nc) electrode with organic/inorganic hybrid nanostructures will undoubtedly improve overall performance of dye-sensitized solar cells (DSSC). Inorganic nc-ZnO is a promising candidate for such hybrid structures, due to its unique properties, such as high conductivity, wide bandgap (3.2 eV), and high excitonic binding energy (60 mV) [1]. In addition, the conduction band edge position of ZnO (-4.3 eV) is similar to that of TiO_2 _(-4.5 eV). Furthermore, ZnO can easily be electrochemically deposited (ECD) at low temperature, and hybrid nanostructures with ZnO can also be co-deposited with dyes such as Eosin-Y (EoY). It has been reported that the dye molecules are strongly attached to the ZnO matrix, filling the voids by means of sulfonic or carboxyl groups [2]. In this article, we report on a study of different hybrids of ZnO nanostructures obtained by changing the dye in the deposition electrolyte. While different aspects of pure ZnO have been reported extensively by the other authors [3,4], this article focuses on the growth of various ZnO porous structures with dyes and their use in energy conversion.

### Experimental details

The ECD of nc-ZnO was carried out in a three-electrode cell consisting of a cathode [substrate, indium oxide (ITO)-coated glass with a ~10 Ω/□ sheet resistance], a Pt counter electrode, and an Ag/AgCl reference (+222 mV vs normal hydrogen electrode). Three ECD baths were prepared, each solution containing a mixture of 0.1 M KCl (Merck) and 5 × 10^-3 ^M ZnCl_2 _(Merck). The first electrolyte bath contained 1 × 10^-4 ^M of EoY (Sigma Aldrich, Spain), while the other baths had two different concentrations of tetrasulfonated copper phthalocyanines (TS-CuPc) (1 × 10^-4 ^M and 3 × 10^-5 ^M). The optimum EoY concentration for ZnO/EoY was derived during a previous study of EoY concentrations [5]. The electrolytes were purged with O_2 _at a volume flow rate of 200 mL/min, while stirring by means of a magnetic bar stirrer to facilitate the oxygen diffusion. Before the deposition process, the ITO glass was ultrasonically cleaned in acetone and subsequently with ethanol for 15 min, and then rinsed with deionized water. The total deposition time for ZnO/Ts-CuPc (approx. 1 μm thick), was 600-800 s, while for ZnO/EoY films, it was 200-500 s. The potentiostatic deposition was carried out by applying a potential of -0.9 V to the substrate (1.17 cm^2^), using an Autolab potentiostat. The bath temperature was set at 70°C controlled by a thermostat. After deposition, the ZnO/EoY films were immersed in a dilute aqueous NaOH solution (pH 10.5) for 40 min to desorb the loaded EoY molecules, while the ZnO/TS-CuPc films were immersed for 24 h. The films were dried in air for 1 h at 150°C. Desorbed thin films were re-sensitised with the relevant dye concentrations for characterization.

### Characterization

The morphology of the ZnO/organic hybrid films was studied using a JEOL-JSM6300 scanning electron microscope (SEM) operating at 10 kV. The structural characterization was carried out by high-resolution X-ray diffraction (XRD) using a Rigaku Ultima IV diffractometer in *θ*-2*θ *mode with a copper anticathode (CuKα, 1.54 Å). Optical transmittance measurements were performed by means of an Ocean Optics DT-MINI-2-GS deuterium-halogen lamp in association with a 500-mm spectrometer coupled to a backthinned CCD detector optimized for the UV-Vis range. To determine the surface topography, an atomic force microscope (AFM) Multimode Veeco was used, where the scanning was carried out in tapping mode using a silicon cantilever. The scanning frequency was set at 0.5 Hz, and the image size was 5 × 5 μm^2^. The photoelectrochemical study was performed in a conventional three-electrode arrangement in a glass cell, consisting of the deposited thin film as the working electrode, illuminated from the glass/ITO side, a Pt counter electrode, and an Ag/AgCl reference electrode in 0.1 M KCl electrolyte. The photocurrent was measured using a potentiostat/galvanostat and recorded. The illumination time of the electrode was controlled using an automatic mechanical shutter, with an adjusted illumination time of 10 s, for which a controller box had been designed. The shutter required approximately 10 ms to reach a completely open (or closed) position. All the measurements were performed at 0.05 V bias where the dark current was negligible.

## Results

### AFM analysis of nanostructured ZnO films

**Figure 1:**
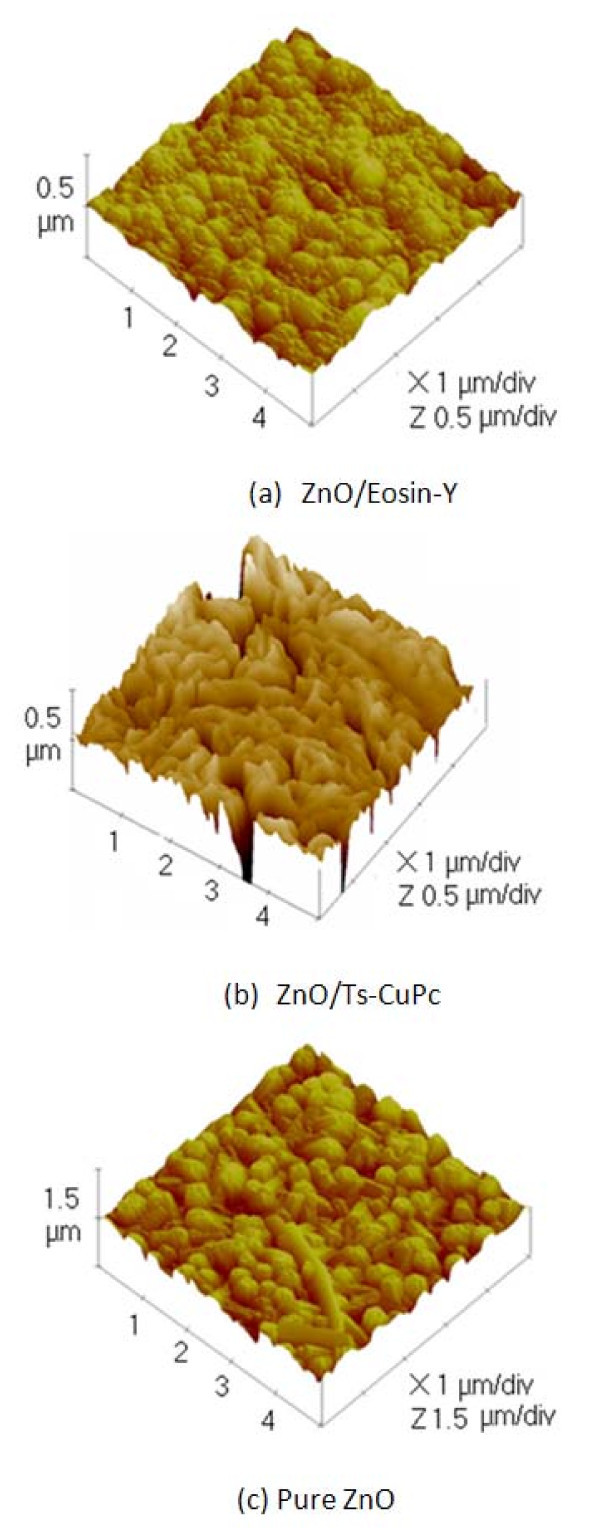
**AFM images of nanostructured hybrid ZnO thin films**.

Figure 1 shows AFM images of the surface topography of the nanostructured hybrids of ZnO with (a) EoY, (b) TS-CuPc, and (c) ZnO without dyes. The pure ZnO (Figure 1c) is composed of large crystals (about approx. 500 nm diameter), suggesting high crystallinity of the film. The conductivity of ZnO films is high enough to allow for an efficient redox process, which also provides a large internal surface available for dye intercalation. The ZnO/EoY sample (Figure 1a) shows domains of the same size or even larger than pure ZnO, consisting of numerous features with the size of a few hundred nanometers. This structure appears to show mesoporous morphology with open pores formed during the exposure to NaOH. The pores are undetectable in our topography measurements, because tips with large curvature radii (approx. 30 nm) were used. Ts-CuPc particles were absorbed by the irregularly shaped ZnO. On the other hand, in ZnO/EoY hybrid films, a formation of clusters of EY^4-^/Zn^2+ ^was observed, in which the edges of the particles were indistinct with a granular and compact morphology. In addition, some cracks on the surface of the hybrid film were seen. Subsequently, globular ZnO clusters were observed, connected one to another, and assimilated within a porous structure. Furthermore, as Figure 1b shows, the structures with TS-CuPc dye are found to be of non-uniform morphology consisting of open and closed pores.

### Surface morphology with SEM

**Figure 2:**
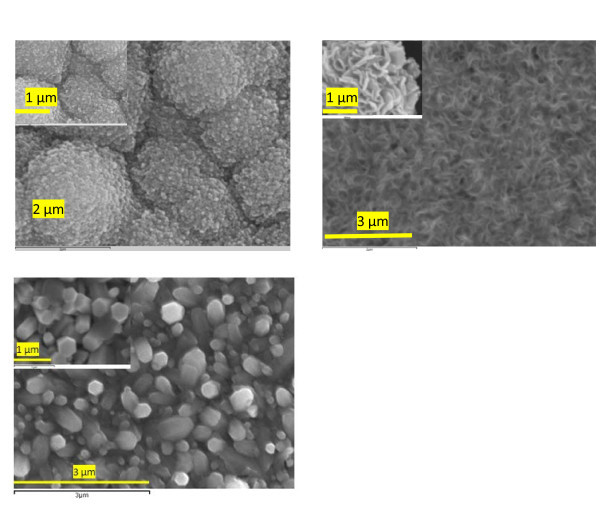
**SEM images of ZnO/Dye hybrid thin films**.

Figure 2 shows the SEM images of deposited ZnO/Dye hybrid thin film structures. The surface morphology of the ZnO/EoY hybrid films shows irregularly connected nanoparticles randomly stacked in large domains (approx. 1 μm), forming a porous structure. As Figure 2a shows each particle domain is made up of an aggregation of small nanoscale crystallites. A highly porous structure would increase the surface area of ZnO significantly. In contrast, the morphology of ZnO/TS-CuPc is cauliflower-like with a porous structure (Figure 2b) consisting of a few nanometer-thick vertically aligned ZnO nanoflakes. These elongated nanoflakes are deeper compared with the structure of the film of ZnO/EoY, and the discontinuity between nanoflakes makes a significantly porous structure. Both these structures are different from that of pure uniform hexagonal phase ZnO (Figure 2c); however, for the optimum efficiency of the ZnO structure, the dye should form a unimolecular layer on the porous surface of ZnO film covering the entire surface. EoY dye would have been completely desorbed from the hybrid film when the film was soaked in NaOH, and hence it was soaked for only 40 min. The ZnO/TS-CuPc films could not be completely desorbed, even after 24 h, as these films consist of both closed and open pores, while ZnO/EoY films only contain open pores.

Desorption of dye can be easily followed using the naked eye because the color of the films varies according to the dye concentration. Furthermore, desorption of dye in the films can also be assessed by energy dispersive X-ray analysis through the Cu/Zn or Br/Zn ratios. For the ZnO/Ts-CuPc hybrid films with a concentration 5 × 10^-3 ^M, the ratio Cu/Zn was 0.008%, whereas in desorbed thin films, it was 0.002%. This shows that only 25% of the dye in the hybrid film is maintained after desorption.

### Optical absorbance

**Figure 3:**
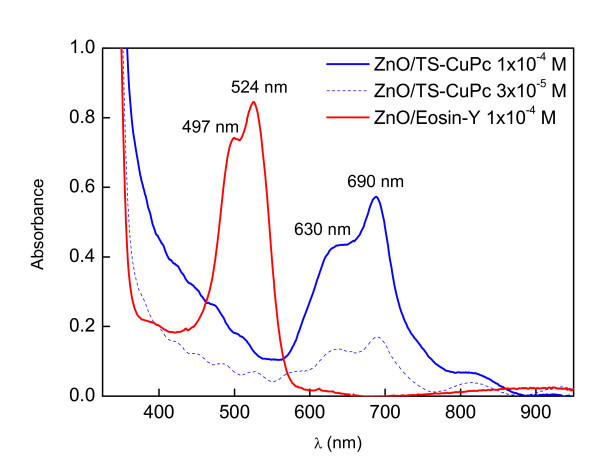
**Absorption spectra of the as-deposited hybrid thin films**.

The absorbance spectra of the ZnO/dye hybrid thin films, as shown in Figure 3, include peaks located in the visible part of the solar spectrum (400-575 nm) with an absorption maximum at 524 nm and a secondary peak at 497 nm, as observed previously [6]. In the case of ZnO/TS-CuPc, both spectra of two different concentrations (5 × 10^-3 ^M and 1 × 10^-4 ^M) show a broad absorption band from 570 to 730 nm with a maximum absorption peak at 690 nm (porphyrin Q-bands). This broadening of the porphyrin Q-band with increased concentration indicates the formation of strongly coupled dye aggregates [7]. From the solution containing TS-CuPc sensitizers, the dye was found to be adsorbed to the growing ZnO. The adsorbed dye molecules are only loosely bonded, which explains the formation of a high density of large diameter pores during NaOH treatment, as observed in the morphology analysis. The spectral characteristics in the uptake of TS-CuPc were so few that even the weak porphyrin Q-bands dominated that part of the spectrum. Increased dye concentration also clearly increased absorption.

### X-ray diffraction

**Figure 4:**
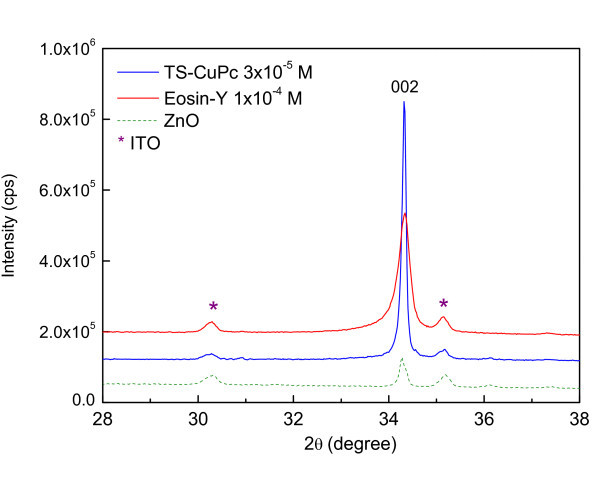
**XRD spectra of pure ZnO and ZnO/dye hybrid films**.

Figure 4 shows the effect of dye on the XRD pattern of ZnO films and hybrid films of ZnO/EoY and ZnO/TS-CuPu. In general, the pure ZnO and the ZnO/hybrid structures all showed a highly textured (002) hexagonal wurtzite structure. It is known that such films have a preferential orientation with the *c*-axis parallel to the substrate plane, consistent with the upright hexagonal columns, as shown in the bird's eye view of pure ZnO in the SEM image in Figure 2c. The relative peak intensities of the XRD spectra as shown in Table 1 confirm the preferential (002) orientation of all the three samples. Unlike in cases like those of Liu et al. [8] who reported the change in crystalline orientation of ZnO following dye adsorption, the crystallographic orientation of ZnO films remains unchanged by the dyes. However, there is a sharp contrast in the relative peak intensities between the pure ZnO and ZnO/dye hybrid thin films. Both the hybrid structures showed improved crystallinity compared with pure ZnO, as confirmed by the improved average crystalline domain size estimated from Scherrer's formula, corrected for stress-related and instrument-related broadenings for large grain (>100 nm) structures, using the full width at half maximum (FWHM) of each spectrum [9,10]. Although the decrease in the FWHM of the spectra for ZnO/Dye films was observed, and hence the improvement of grain sizes in comparison with that of pure ZnO, the improvement in crystallinity was more prominent in the ZnO/TS-CuPc hybrid thin films than in the ZnO/EoY. The average crystalline domain size for ZnO/TS-CuPc structure was approx. 4.5 nm, while that of ZnO/EoY was approx. 1.5 nm.

**Table 1:** Relative peak intensities of three XRD spectra shown in Figure 4

*d*(*hkl*)	Relative peak intensity		
	
	ZnO (Green)	EoY (Red)	CuPc (Blue)
(002)	1	1	1
(101)	0.10	0.002	0.013
(100)	0.05	0.007	0.008

### Photocurrent analysis

**Figure 5:**
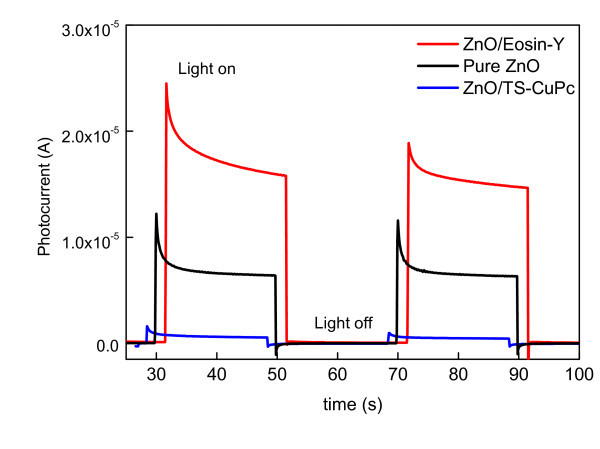
Photocurrent measured **at one-step electrodeposited pure ZnO (black) and hybrid thin films under white light illumination**.

Despite inferior crystallinity (to ZnO/TS-CuPc), ZnO/EoY provides an efficient route for transport of electrons with a relatively high photocurrent response (Figure 5). However, the maximum efficiency obtained for ZnO/EoY hybrid structures when applied in DSSCs is only 2.3% [11] despite the closely matched energy levels between EoY and ZnO. Theoretical analyses confirm that this low efficiency is because only a small fraction of the electrons generated in the highest occupied molecular orbital of the dye has sufficient energy to transfer to the lowest unoccupied molecular orbital [12]. The photocurrent of the ZnO/TS-CuPc thin films was insufficient, as shown in Figure 5, due to the mismatching of energy levels between TS-CuPc and ZnO. This dye adsorption occurs mostly between stacked ZnO layers of the mesoporous structure, masking the dye from absorbing any light and, therefore, generating no photocurrent.

ZnO/EoY photoresponse showed an overshoot at the incidence of light, characteristic of a slow regeneration reaction [13] of the oxidized dye molecule following fast injection to ZnO, which is usually caused by non-exposure of dyes due to masking by the electrolyte. This non-exposure of dyes to light leads to a steady-state concentration of oxidized dye suggesting the presence of a positively charged interface, an increased recombination of charge carriers, and hence a small photocurrent. When the light is switched off, this recombination is directly observed in a small cathodic spike, suggesting the discharge of the positive surface charge by the electrode [14]. For ZnO/TS-CuPc, the characteristics are similar, but the initial overshoot, as well as the cathodic current, as smaller in magnitude, are due possibly to deep traps in the ZnO. The overshoot can also be caused by the absorption of a fraction of light on the film surface. These observations confirm poor hybridization of TS-CuPc with ZnO, which results in poor efficiency in the generation of electrons. However, EoY appears to show formation of strong hybrid mesoporous structures with the ZnO.

## Conclusions

Changes in the morphology of the ECD ZnO were studied using two sensitizer dyes, EoY and TS-CuPc. Ts-CuPc particles absorbed by the ZnO were found to be irregularly shaped. On the other hand, in ZnO/EoY hybrid films, the EY^4-^/Zn^2+ ^were observed forming clusters in which the clear edges of the particles were absent, showing a granular and compact morphology with few cracks on the surface of the hybrid film. The crystallinity improved when dyes of ZnO were used, while maintaining the preferred orientation of ZnO in the direction (002). ZnO/TS-CuPc showed better crystallinity despite a large porous structure in comparison with ZnO/EoY, as shown by the average crystalline sizes. The photocurrent response indicates that a high proportion of dye molecules are being excited by the electrons acting as sensitizers. However, the absorbance peak of two dyes (524 and 690 nm), proves that the two dyes could be used together to improve DSSC efficiency.

## Abbreviations

AFM: atomic force microscope; DSSC: dye-sensitized solar cells; ECD: electrochemically deposited; EoY: Eosin-Y; FWHM: full width at half maximum; ITO: indium oxide; nc: nanocrystalline; SEM: scanning electron microscope; TS-CuPc: tetrasulfonated copper phthalocyanines; XRD: X-ray diffraction.

## Competing interests

The authors declare that they have no competing interests.

## Authors' contributions

MoM and MiM with BM carried out the nc-ZnO sample fabrication, materials analysis and interpretation of the results. BM and APS participated in photocurrent measurements, analysis, interpretation of the results and also coordinated the collaborations. All authors read and approved the final manuscript.
